# Integrative omics connects N-glycoproteome-wide alterations with pathways and regulatory events in induced pluripotent stem cells

**DOI:** 10.1038/srep36109

**Published:** 2016-11-03

**Authors:** Putty-Reddy Sudhir, Madireddy Pavana Kumari, Wei-Ting Hsu, Chein-Hung Chen, Hung-Chih Kuo, Chung-Hsuan Chen

**Affiliations:** 1 Genomics Research Center, Academia Sinica, Taipei 11529, Taiwan; 2 Institute of Cellular and Organismic Biology, Academia Sinica, Taipei 11529, Taiwan

## Abstract

Molecular-level differences ranging from genomes to proteomes, but not N-glycoproteomes, between human induced pluripotent stem cells (hiPSCs) and embryonic stem cells (hESCs) have been assessed to gain insights into cell reprogramming and induced pluripotency. Our multiplexed quantitative N-glycoproteomics study identified altered N-glycoproteins that significantly regulate cell adhesion processes in hiPSCs compared to hESCs. The integrative proteomics and functional network analyses of the altered N-glycoproteins revealed their significant interactions with known PluriNet (pluripotency-associated network) proteins. We found that these interactions potentially regulate various signaling pathways including focal adhesion, PI3K-Akt signaling, regulation of actin cytoskeleton, and spliceosome. Furthermore, the integrative transcriptomics analysis revealed that imperfectly reprogrammed subunits of the oligosaccharyltransferase (OST) and dolichol-phosphate-mannose synthase (DPM) complexes were potential candidate regulatory events for the altered N-glycoprotein levels. Together, the results of our study suggest that imperfect reprogramming of the protein complexes linked with the N-glycosylation process may result in N-glycoprotein alterations that affect induced pluripotency through their functional protein interactions.

Somatic cell reprogramming technology has been introduced to generate embryonic stem cell (ESC)-like cells known as induced pluripotent stem cells (iPSCs)^1^,^2^. Since the introduction of *in vitro* generated human iPSCs (hiPSCs)^1^,^3^, iPS cell-based therapy has become one of the major interests of clinical investigators because hiPSCs avoid the ethical issues associated with the use of human embryos. Besides, iPSCs can be used as a potential source for drug screening, disease modeling, and the development of cell-based therapeutics^4^,^5^. However, pluripotency reprogramming causes genetic and epigenetic alterations in iPSCs that may result in an increased risk of neoplasms^6^,^7^. In addition, it has been reported that the current pluripotency reprogramming procedure causes alterations at the molecular level (e.g., genes, proteins, post-translational modifications, and metabolites) in iPSCs as compared to the ESCs^8^,^9^. These alterations potentially affect the functional characteristics, such as the self-renewal and differentiation potential (i.e., pluripotency), of the iPSCs. Therefore, it is essential to gain insights into the reprogramming process and induced pluripotency by exploring the differences between ESCs and iPSCs^8^,^9^,^10^ at the molecular level to improve the quality of iPSCs for basic research and to implement safer and effective iPS cell-based therapies.

Although hiPSCs and hESCs exhibit similar characteristics such as their morphologies in culture, growth requirements, expression of pluripotency-associated markers and genes, and *in vitro* and *in vivo* developmental propensity, our understanding of their similarity on molecular level is still elusive^8^,^9^,^10^,^11^. In recent years, several high-throughput studies have demonstrated various similarities and differences between hiPSCs and hESCs at the molecular level. These studies include analyses of genetic and epigenetic profiles^7^,^12^,^13^,^14^, microRNA profiling^15^,^16^, gene expression analyses using transcriptomics and proteomics approaches^17^,^18^,^19^,^20^,^21^, posphoproteomics profiling^20^, and metabolome profiling^22^. Some of these studies have reported that the observed differences between the hiPSCs and hESCs are lab-specific, but others have attributed the differences to parental somatic memory, stress during reprogramming, and adaptation to the culture conditions^8^,^9^,^10^. Recently, a cell surface N-glycoproteome study has revealed several markers, epitopes, and drug targets using human pluripotent stem cells (hPSCs (hiPSCs and hESCs)) and somatic cells (SCs)^23^. The transcriptome and surface proteome data were integrated to compare the cells. However, it has not been explored how similar the hiPSCs and hESCs are at the N-glycoproteome level.

In this study, we reported the N-glycoproteomic signatures of multiple cell lines (five hiPSCs, two hESCs, and two hiPSC parental SCs) using an N-glycoproteomics approach. Multiplexed quantitation of these signatures identified cell type-specific and cell general alterations of N-glycoprotein expression in hiPSCs. Furthermore, using integrative proteomics and protein interaction network analyses, we found that altered N-glycoproteins regulate the functions of PluriNet (pluripotency-associated network) proteins in various signaling pathways. In addition, an integrative transcriptomics analysis explored the imperfectly reprogrammed subunits of the protein complexes that are potentially responsible for the N-glycoprotein alterations observed in hiPSCs. These novel results provide a basis for future studies on strategies to improve the reprogramming efficiency and induced pluripotency of hiPSCs in the context of the post-translational protein N-glycosylation.

## Results

### N-glycoproteomic profiling of hiPSCs, hESCs, and parental SCs

**Figure 1:**
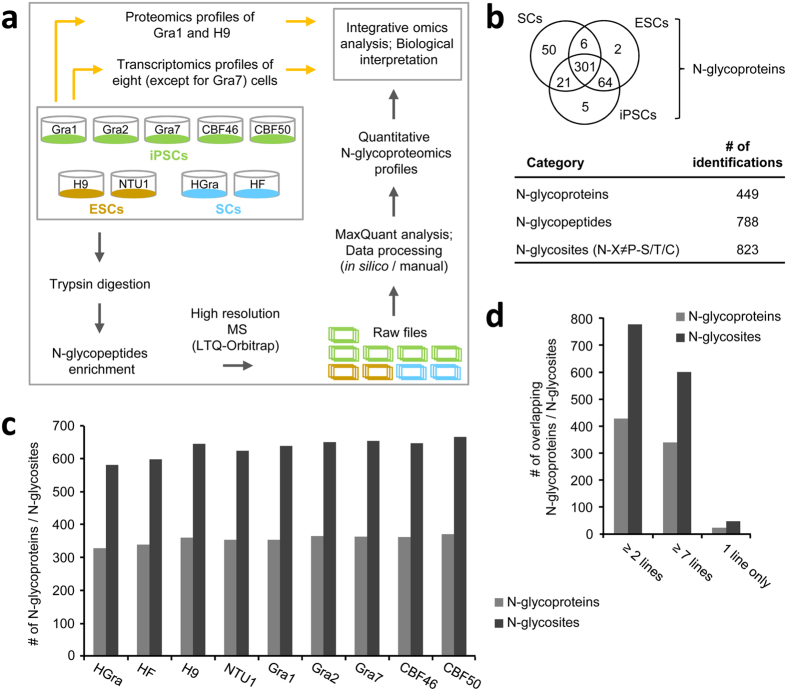
Schematic workflow of the experimental design and summary of the N-glycoproteomics results. **(a)** N-glycopeptides were enriched from nine cell lines (five hiPSCs, two hESCs, two parental somatic cells (SCs)) using the hydrazide chemistry approach, and were analyzed in triplicates using LTQ-Orbitrap mass spectrometry (MS). The raw MS files were processed by MaxQuant and the resulting N-glycoproteomics profiles were quantitatively compared using *in silico* and manual data analyses. Furthermore, the N-glycoproteomics profiles were integrated with the proteomics profiles (Gra1 and H9 Cells) and transcriptomics profiles (except for the Gar7 cells) to explore the biological significance of this study. **(b)** The Venn diagram represents the overlap between the N-glycoproteins identified in the nine cell lines from the three groups. In addition, the total numbers of N-glycoproteins, N-glycopeptides, and N-glycosites identified are shown. **(c)** The total numbers of N-glycoproteins and N-glycosites identified in each of the nine cell lines are shown. **(d)** The total numbers of N-glycoproteins and N-glycosites that were identified in any one of the cell lines, overlapped in 2 or more cell lines, and overlapped in 7 or more cell lines are shown.

Our previous studies reported the derivation and characterization of hiPSCs from granulosa (HGra) and fibroblast (HF) cells using the pluripotency reprogramming approach^24^,^25^. In the present study, we focused on the N-glycoproteomic profiles of multiple hiPSCs, hESCs, and hiPSC parental somatic cells (SCs) to explore the induced pluripotency and cell reprogramming process. Figure 1a shows the detailed workflow of the label-free quantitative N-glycoproteomic analysis and the integrative omics analysis. Briefly, we used a total of nine cell lines, which include five hiPSC (Gra1, Gra2, Gra7, CBF46, and CBF50), two hESC (H9 and NTU1), and two hiPSC parental SC (HGra and HF) lines. Three (Gra1, Gra2, and Gra7) hiPSC lines were derived from HGra cells and the other two lines (CBF46 and CBF50) were derived from HF cells. First, the proteins from these nine cell lines were extracted, denatured, and digested with trypsin. Then, the N-glycopeptides were enriched from the trypsin-digested samples using the hydrazide chemistry approach, and analyzed using high resolution mass spectrometry (MS). All nine biological samples were analyzed in triplicates and the raw data files obtained from MS were processed using a MaxQuant analysis^26^,^27^. The MaxQuant analysis not only identifies the site-specific N-glycosylation events but also quantitates their expression levels in multiple samples.

Figure 1b shows a summary of identification results of the N-glycosylation events obtained from MaxQuant analysis. A total of 449 N-glycoproteins were identified from the nine cell lines (five hiPSCs, two hESCs, and two SCs). Of the 449 N-glycoproteins, 365 showed overlap between two types of pluripotent stem cells (hPSCs (hiPSCs and hESCs)) and 301 showed overlap between three types of cells (hiPSCs, hESCs, and SCs) (Fig. 1b, Supplementary Table 1). Interestingly, we identified alkaline phosphatase, a well-known marker of PSCs^23^, in hiPSCs and hESCs but not in SCs. A previous study has reported several N-glycoprotein markers of both mouse and hPSCs or hPSCs only^23^. Consistent with the previous study^23^, our study only identified AMIGO3, FLT1, GABRB3, IGSF1, LAMA1, LINGO1, LRRN1, NLGN4X, and SEMA4B in hPSCs. Furthermore, we only identified a negative marker (FBN1) of hPSCs in SCs, and the Thy1 (CD90) protein marker was observed in hPSCs and SCs, as expected^23^. Moreover, we identified a total of 788 N-glycopeptides and 823 N-glycosites with a conserved sequence (N-X≠P-S/T/C) corresponding to the 449 N-glycoproteins (Fig. 1b, Supplementary Table 1). Threonine (n = 512) was identified at a higher frequency than serine (n = 308) and cysteine (n = 3) residues at the second position of the conserved sequence, as previously described^28^,^29^. Furthermore, we analyzed the numbers of N-glycosylation events identified in each cell line and multiple cell lines. We found that there were no large differences in the numbers of N-glycoproteins and N-glycopeptides identified across nine cell lines, particularly in case of the hPSC lines (Fig. 1c). In addition, a small number of N-glycosites or N-glycopeptides were identified in any one of the nine cell lines, and thus we could not compare their expression levels with the other cell lines. However, most of the events were identified in two or more cell lines (Fig. 1d), which is compatible for the quantitative analysis.

### Assessment of the quality of the N-glycoproteomic profiles from hiPSCs, hESCs, and parental SCs

Next, we examined the LC-MS/MS performance and the quality of the N-glycoproteomics dataset. First, we assessed the correlation between the N-glycopeptide expression levels among three replicates for each cell line. An excellent correlation (r = 0.939–0.968) between the triplicate analyses was observed in all of the nine cell lines (Supplementary Fig. 1a), suggesting a good reproducibility of the LC-MS/MS (LTQ-Orbitrap) analysis. Second, as expected, we observed a higher correlation in comparisons of similar types of cell lines (hiPSCs:hESCs (r = 0.784–0.926)) than in different types of cell lines (hiPSCs/hESCs:SCs (r = 0.407–0.584)), indicating that the N-glycopeptides expression levels are highly different between SCs and hiPSCs/hESCs, and poorly or moderately different between hiPSCs and hESCs (Supplementary Fig. 1a). This correlation analysis was performed using the data from nine cell lines. Third, we plotted the number of N-glycopeptides identified in three or two replicate analyses or in one replicate analysis of each cell line to examine the performance of the LTQ-Orbitrap in replicate analyses. In the nine cell lines, the majority of the N-glycopeptides were identified in three (81.96–87.03%) or two (7.17–10.95%) replicate analyses (Supplementary Fig. 1b). As expected, a small percentage (4.3–7.96%) of N-glycopeptides was identified in one replicate analysis. Finally, we assessed the quality of the data by measuring the coefficient of variation (CV) of the N-glycopeptide expression levels in replicate analyses. A majority (71.1–83.03%) of the N-glycopeptide expression levels were identified as having a ≤20% CV in the nine cell lines (Supplementary Fig. 1c). These results indicate the quality of the N-glycoproteomics dataset for the nine cell lines obtained from the MS analysis.

### Multiplexed label-free quantitation identifies hiPSC type-specific and hiPSC general N-glycoprotein alterations

**Figure 2:**
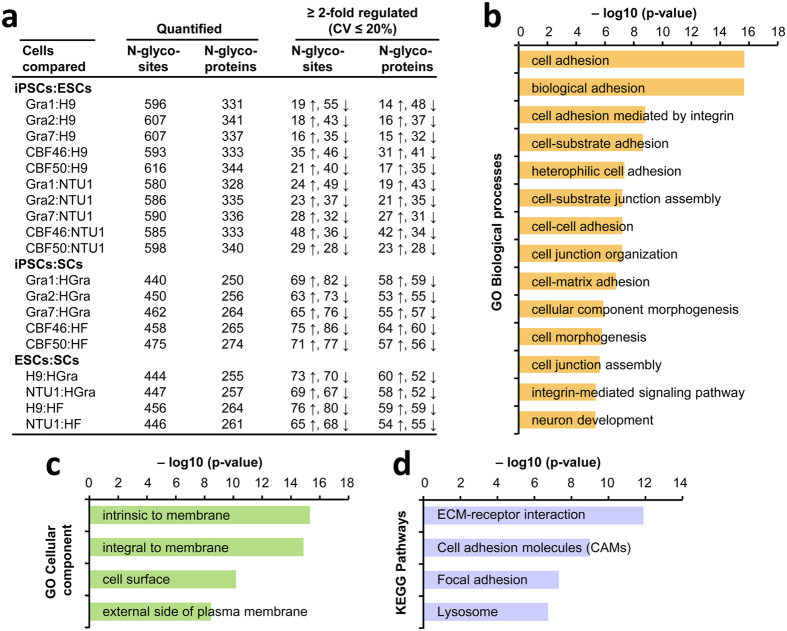
The hiPSC type-specific N-glycoprotein alterations and the regulated biological processes and signaling pathways. **(a**) The table shows the hiPSC type-specific N-glycoprotein alterations identified in 19 quantitative comparisons of hiPSCs and hESCs as well as PSCs (hiPSCs or hESCs) and SCs. The numbers of comparable and differentially regulated (≥2-fold, and CV ≤ 20%) N-glycoproteins and N-glycosite-specific events are shown. The most significant biological processes **(b**) cellular components **(c)** and signaling pathways **(d)** linked with the altered N-glycoproteins identified in hiPSCs compared to hESCs are shown. The biological processes and cellular components are Gene Ontology terms whereas the signaling pathways are KEGG terms. These terms were enriched with the significance (p-value) < 0.0001 and a false discovery rate (FDR) < 0.01 using the DAVID database.

We performed a total of 19 label-free quantitative comparisons across the nine cell lines (Fig. 2a and Supplementary Table 2) to identify the N-glycoprotein alterations. Nine of the 19 comparisons were performed between PSC lines (five hiPSCs/two hESCs) and two SCs using comparable N-glycoproteins (n = 250–274) and N-glycopeptides (n = 440–475). These comparisons revealed that an average of 43.2% of N-glycoproteins exhibited ≥2-fold differences (CV ≤ 20%) between PSCs and SCs (Fig. 2a and Supplementary Table 2). A large percent difference was observed between the PSC and SC lines because of their very different genetic backgrounds. We observed the highest percentage of 2-fold differences between hiPSC Gra1 and HGra cells (46.4%) and the lowest percentage for the hiPSC CBF50 and HF cells (40.5%). The other 10 of the 19 comparisons were performed between two types of PSCs (five hiPSCs and two hESCs) using 328–344 N-glycoproteins and 580–616 N-glycopeptides. This analysis revealed that an average of 17.1% of N-glycoproteins exhibited ≥2-fold differences (CV ≤ 20%) between the hiPSCs and hESCs. We observed the highest percentage of 2-fold differences between CBF46 and NTU1 cells (21.6%) and the lowest percentage between the hiPSC Gra7 and hESC H9 cells (13.6%). In agreement with the clustering analysis (Supplementary Fig. 1a), in which the three types of cells fell into two main categories (hPSCs (five hiPSCs/two hESCs), and two SCs), the lower percentage of differences observed between the hiPSCs and hESCs relative to hPSCs and SCs was attributed to their similar types of N-glycopeptide expression levels. The ten comparisons performed between the hiPSCs and hESCs revealed a total of 164 unique hiPSC type-specific N-glycoproteins with ≥2-fold alterations (56 up-regulated, 72 down-regulated, and 36 with both up- and down-regulated N-glycosylation events). We subjected these 164 candidate proteins to a functional annotation tool, the DAVID database, to study the functional significance. The DAVID analysis revealed the Gene Ontology biological processes and cellular components (Fig. 2b,c and Supplementary Table 3) associated with these candidates. We found that the candidates are predominantly located in membrane regions and mainly involved in cell adhesion processes. In addition, the candidates were associated with signaling pathways that regulate cell adhesion processes (Fig. 2d and Supplementary Table 3).

We next focused on the 10 quantitative comparisons performed between five hiPSC and two hESC lines to identify the hiPSC general N-glycoprotein alterations. The N-glycoproteins that were identified as being up- or down-regulated by 2-fold in at least 5 of the 10 comparisons were considered as hiPSC general N-glycoprotein alterations (Supplementary Fig. 2a). A total of 34 N-glycosites corresponding to 32 N-glycoproteins were identified in this category and were listed in Supplementary Fig. 2b, along with their site-specific N-glycopeptides and observed fold-changes. Of the 32 N-glycoproteins, eight and 23 were identified as being up-and down-regulated, respectively, in hiPSCs compared to hESCs. The other protein consists of two N-glycopeptides, where one was up-regulatied 2-fold and the other one was down-regulated 2-fold in hiPSCs. Most interestingly, EGFR, GGT1, HYOU1, ITGA5, ITGAV, KIAA0319L, PLOD3, and SLC2A3 showed 2-fold differences in at least 8 of the 10 comparisons (Supplementary Fig. 2b). We subjected these 32 candidate proteins to the DAVID database to study the functional significance. The DAVID analysis revealed that more than two-thirds of the 32 proteins (81%) were located intrinsic or integral to the membrane and more than one-third (37%) were involved in cell adhesion or biological adhesion processes (Supplementary Fig. 2c). The proteins specific to localization and adhesion processes were listed in Supplementary Fig. 2c. This analysis suggests that several candidate N-glycoproteins are closely associated, based on their localization and roles in regulating the cellular processes and molecular functions. The same analysis of all N-glycoproteins that showed differences between the hiPSCs and hESCs revealed that 73% of the N-glyocoproteins were located intrinsic or integral to the membrane and 22% were associated with cell adhesion or biological adhesion processes (Supplementary Table 3). These quantitative N-glycoproteomics results from multiple cell lines would serve as a resource for the stem cell research community to understand hiPSC type-specific N-glycoprotein signatures. In addition, the identified hiPSC general N-glycoprotein alterations would serve as biomarkers to distinguish them from hESCs.

### An integrative proteomics analysis validates the hiPSC type-specific N-glycoprotein alterations

**Figure 3:**
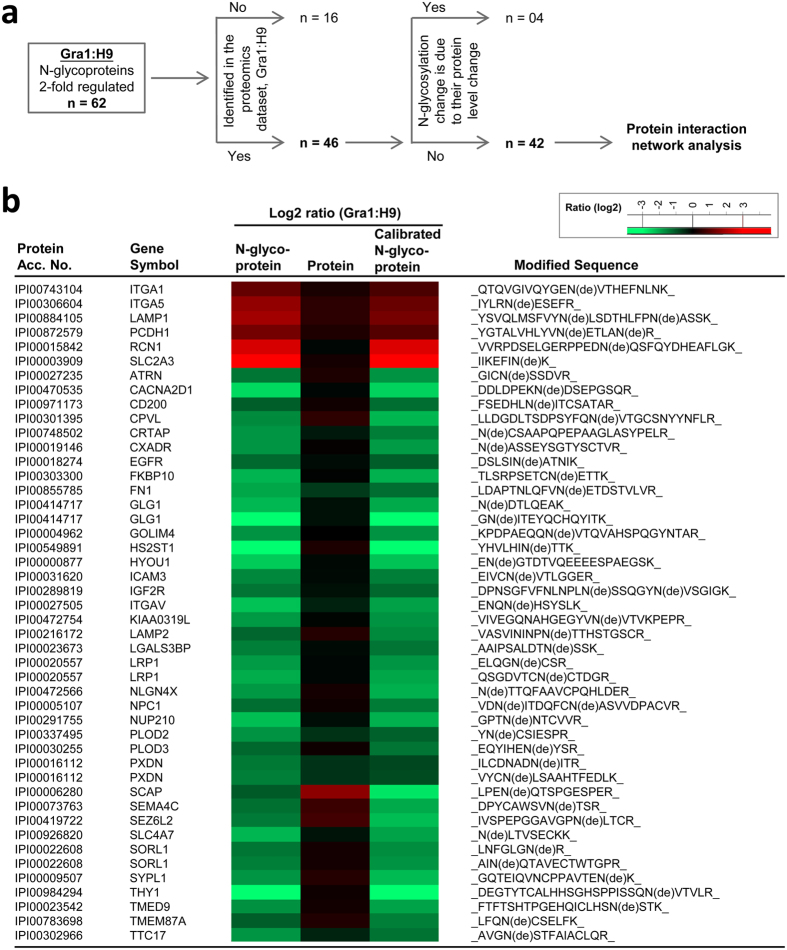
Integrative proteomics-based validation of the hiPSC type-specific N-glycoprotein alterations. **(a)** The integrative approach shows the validation of the hiPSC type-specific N-glycoprotein alterations (n = 62) identified in Gra1 cells (hiPSCs) compared to H9 cells (hESCs) using their corresponding quantitative proteomics data set. Among the 62 altered N-glycoproteins, 46 were calibrated using their protein expression levels, and 42 of 46 were identified as truly altered in Gra1 cells. **(b)** We show the accession numbers (IPI ids), symbols, fold changes before and after calibration, and site-specific N-glycopeptides of the 42 N-glycoproteins represented in Fig. 3a. The color bar represents the fold change values.

Next, we calibrated the altered N-glycoprotein expression levels using their corresponding protein expression levels. This calibration is critical to rule out the possibility that the altered protein expression levels may result in altered levels of post-translational modifications such as protein glycosylation and protein phosphorylation. Therefore, we utilized the proteomics dataset published in our previous study^21^ and attempted to calibrate the 62 altered N-glycoproteins identified between Gra1 hiPSCs and H9 hESCs (Fig. 3a). We identified 46 of the 62 N-glycoproteins in the proteomics dataset and four of the 46 proteins showed N-glycoprotein expression changes due to the changes in their protein levels (Fig. 3a). The 91.3% (42 of 46) of the N-glycoproteins that showed differential regulation after calibration were listed in Fig. 3b. Of the 42 N-glycoproteins, 40 proteins showed ≥ 2-fold changes and the other two (ITGA1 and PXDN) showed at least 1.75-fold changes after calibration. These 42 N-glycoproteins were utilized in the subsequent analysis to assess their functional relationships with pluripotency-associated genes. Our results indicate that the calibration did not result in dramatic changes in the number of altered N-glycoproteins identified in Gra1 hiPSCs because most of the protein expression levels are similar between hiPSCs and hESCs, as reported in previous studies^19^,^20^,^21^.

### The hiPSC type-specifically altered N-glycoproteins regulate signaling pathways through their functional interactions with PluriNet proteins

**Figure 4:**
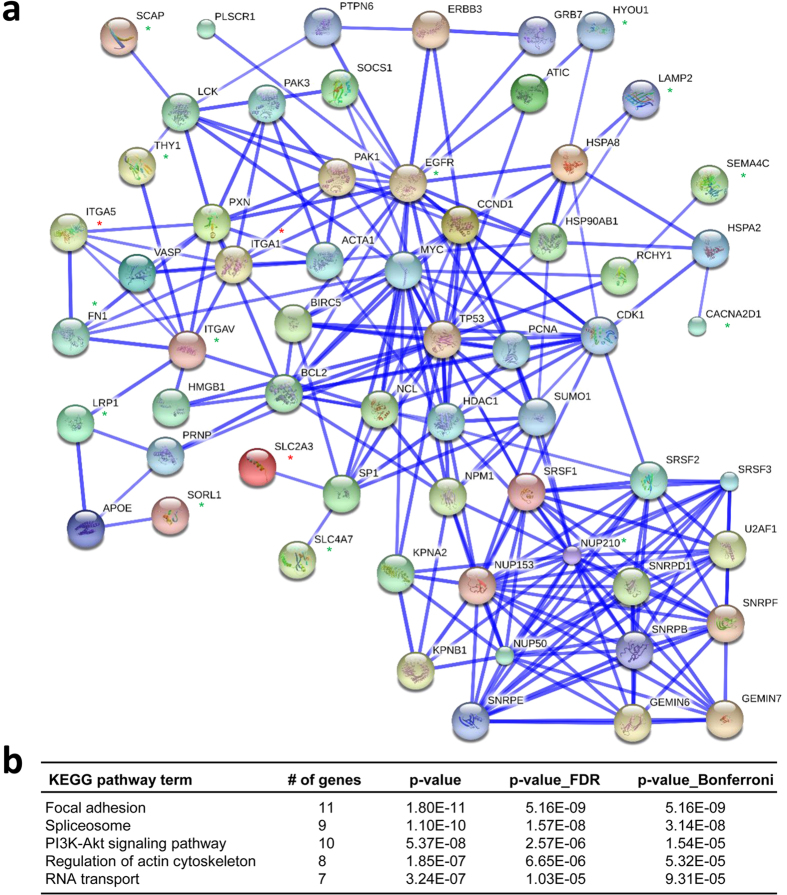
The protein interaction network analysis of hiPSC type-specifically altered N-glycoproteins and PluriNet proteins identifies the functional relationships and regulated signaling pathways. (**a**) Functional relationships between the proteins identified as containing N-glycoprotein alterations in Gra1 hiPSCs and known PluriNet (pluripotency-associated network) proteins are shown. Altered N-glycoproteins are denoted with red (up-regulated) and green (down-regulated) asterisk (*), and the PluriNet proteins are shown without an asterisk. The altered N-glycoproteins that showed at least one direct and high confidence relationship with PluriNet proteins were considered to generate the subnetwork in the STRING database. The subnetwork matrices (coefficient clustering and mean shortest path (p < 0.001)) are significant compared to the 1,000 randomized networks. (**b**) The list of KEGG pathways that was enriched in subnetwork is shown, along with the corrected p-values.

Having identified a compendium of hiPSC type-specific alterations in N-glycoprotein expression, we assessed whether these alterations were functionally interacting with the pluripotency-associated genes. We used the STRING (Search Tool for the Retrieval of Interacting Genes/Proteins) database to examine the functional protein interaction network of altered N-glycoproteins. The STRING database is useful for identifying the physical and functional interactions among the set of genes/proteins of interest^30^. We utilized a set of 299 pluripotency-associated network (PluriNet) genes that were identified in a microarray-based gene expression study of about 150 cell lines^31^ to investigate the roles of the N-glycoprotein alterations in the context of pluripotency. Using the STRING database, we established a functional network between the 42 candidate proteins that showed the N-glycoprotein alterations after calibration (Fig. 3) and the known PluriNet proteins. Furthermore, we considered only the candidates that showed at least one direct interaction with PluriNet proteins to construct the subnetwork (Fig. 4a). This analysis revealed significant functional associations between 16 altered N-glycoprotein candidates and 45 PluriNet proteins (p < 0.001, Fisher’s exact test). This study is the first to report the functional relationship between the altered N-glycoproteins identified in hiPSCs and PluriNet genes based on a network analysis. By comparing the network to the 1,000 randomized networks, we found that the matrices, coefficient clustering and mean shortest path for our protein network are highly significant (p < 0.001). This result indicates that the protein network relationships are biologically well connected and they might associate with the signaling pathways. We subjected the subnetwork to the KEGG pathways enrichment analysis to gain insights into the functional consequences of these significant protein interactions. The analysis indicated that various signaling pathways were significantly enriched (p < 0.005), including focal adhesion, MAPK signaling, PI3K-Akt signaling, spliceosome, regulation of actin cytoskeleton, and RNA transport pathways (Fig. 4b and Supplementary Table 4). These results indicate that the altered N-glycoproteins potentially affect the roles of pluripotency-associated proteins in diverse signaling pathways through functional protein interactions.

### An integrative transcriptomics analysis reveals the potential events that regulate the altered N-glycoprotein levels in hiPSCs

**Figure 5:**
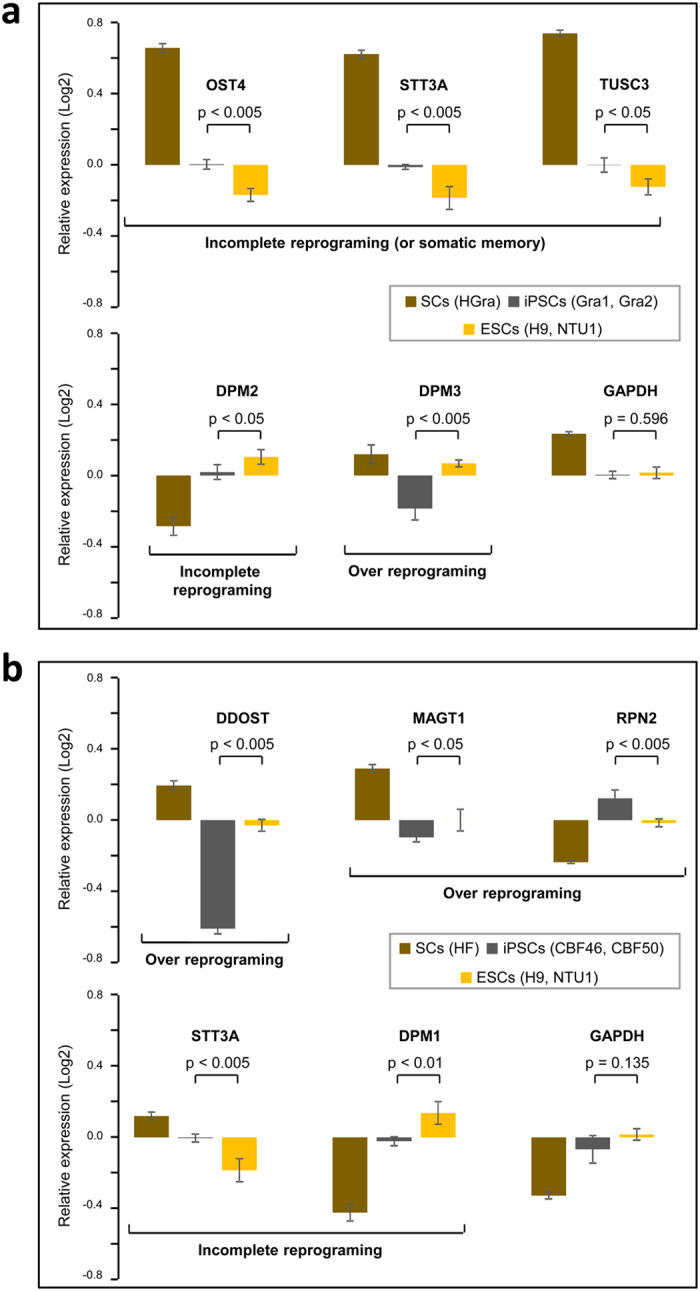
The integrative transcriptomics analysis identifies that imperfect reprogramming of potential regulatory complexes may be responsible for the altered N-glycoprotein levels in hiPSCs. (**a**) Relative gene expression levels of the OST and DPM complex subunits observed in two hESCs (H9 and NTU1), two hiPSCs (Gra1 and Gra2), and SCs (HGra). In addition, the relative expression levels of a housekeeping gene (GAPDH) in these cell lines are shown. The transcriptomics-based gene expression levels were obtained from two biological replicates. The average gene expression levels were derived from replicate analyses and the significant differences between hiPSCs and hESCs were defined using t-test. (**b**) Relative gene expression levels of the OST and DPM complex subunits observed in two hESCs (H9 and NTU1), two hiPSCs (CBF46 and CBF50), and SCs (HF). The other details are the same as described in Fig. 5a.

To understand the molecular causes of the N-glycoprotein alterations observed in hiPSCs, we next examined imperfectly reprogrammed genes using an integrative transcriptomics analysis. Specifically, we assessed the gene expression levels of the central enzyme, oligosaccharyltransferase (OST protein complex), which consists of multiple subunits and is involved in the protein N-glycosylation process^32^,^33^,^34^. To this end, we utilized a previously published transcriptomics data set from eight of the same nine cell lines (except for Gra7) used in this study (Fig. 1a). The results observed in Gra1 and Gra2 hiPSCs, and CBF46 and CBF50 hiPSCs are shown in Fig. 5a,b, respectively. Our analysis revealed significant alterations (p < 0.005–0.05) in the gene expression levels of six subunits (DDOST, MAGT1, OST4, RPN2, STT3A, and TUSC3) of the OST complex between hiPSCs and hESCs (Fig. 5a,b). One of these altered genes (STT3A) is an STT3 protein that functions as the catalytic subunits of the OST complex. Interestingly, we observed significant alterations (p < 0.005) in STT3A in both types of hiPSCs that were derived from different parental somatic cells (Fig. 5a,b). Furthermore, we explored the reprogramming effects on the OST complex subunits by comparing the gene expression levels between hiPSCs, hiPSC parental somatic cells (SCs), and hESCs. The STT3A expression levels were higher in hiPSCs and SCs with respect to the hESCs (hiPSCs > hESCs and SCs > hESCs) (Fig. 5a,b). Similar results were observed for OST4 and TUSC3 (Fig. 5a). This result indicates that incomplete reprogramming resulted in higher levels of OST4, STT3A and TUSC3 in hiPSCs compared to the hESCs. In other words, reprogramming resulted in incomplete silencing (i.e., somatic memory) of OST4, STT3A and TUSC3. The analysis of the DDOST and MAGT1 levels revealed their lower expression in hiPSCs relative to hESCs and SCs (hiPSCs < hESCs and SCs > hESCs). This result indicates that reprogramming has over silenced the DDOST and MAGT1 genes in hiPSCs (Fig. 5b). The RPN2 levels were higher in hiPSCs and lower in SCs relative to hESCs (hiPSCs > hESCs and SCs < hESCs) indicating the over reactivation of RPN2 in hiPSCs (Fig. 5b). These results uncovered the imperfect reprogramming of OST complex subunits in hiPSCs.

Four subunits including a catalytic subunit (OST4, RPN2, STT3A, and TUSC3) of the OST complex were up-regulated, whereas the other two subunits (DDOST and MAGT1) were down-regulated in hiPSCs compared to hESCs (Fig. 5a,b). However, the analysis of hiPSC type-specific and hiPSC general N-glycoprotein alterations showed that there was a larger number of down-regulated N-glycoproteins in the hiPSCs than in the hESCs (Fig. 2a and Supplementary Fig. 2a). Therefore, to further explain the correlation between the down-regulation of N-glycoproteins and imperfectly reprogrammed genes, we searched the literature for other complexes. A protein complex known as dolichol-phosphate-mannose synthase (DPM) with three subunits (DPM1, DPM2, and DPM3) has been identified in human cells^33^. The DPM complex is involved in the enzymatic reactions of the N-glycan biosynthesis process that occurs prior to the protein N-glycosylation process linked with the OST complex. Our analysis revealed a significant down-regulation of DPM3 (p < 0.005) and DPM2 (p < 0.05) expression in Gra1 and Gra2 hiPSCs compared to the hESCs (Fig. 5a). In addition, we observed that DPM1 was down-regulated (p < 0.01) in the CBF46 and CBF50 hiPSCs compared with the hESCs (Fig. 5b). The imperfect reprogramming resulted in incomplete reactivation of the DMP2 and DPM1 subunits (hiPSCs < hESCs and SCs < hESCs) and enhanced silencing of the DPM3 subunit (hiPSCs < hESCs and SCs > hESCs) in hiPSCs (Fig. 5a,b). The reduced expression levels of the DPM complex may contribute to the N-glycoprotein expression changes observed in hiPSCs. These results revealed the imperfect reprogramming of both OST and DMP complexes in hiPSCs.

Next, we asked whether the imperfect reprogramming of the protein complex subunits can be observed in other studies. Therefore, we reviewed the published gene expression profiles of hiPSCs from previous studies. We observed the alterations in the DAD1 and STT3A subunits between multiple hiPSC and hESC lines in an omics study by Phanstiel *et al*.^20^. We also observed alterations in the STT3A and TUSC3 subunits between the hiPSCs generated by episomal vectors and hESCs^35^. In addition, we examined the gene expression meta-analysis data and identified alterations in the DAD1, DDOST, MAGT1, STT3B, and TUSC3 subunits in hiPSCs compared with hESCs^36^. These gene expression results further support the imperfect reprogramming of OST complex subunits in the hiPSCs.

## Discussion

**Figure 6:**
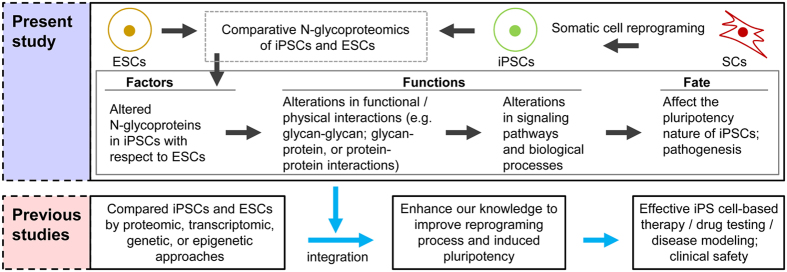
Potential implications of the current study for improving the somatic cell reprogramming and induced pluripotency of hiPSCs.

Altered post-translational modifications such as protein phosphorylation or glycosylation regulate the pluripotency of ESCs^37^. In particular, abnormalities in glycoprotein expressions alter the protein-protein, protein-carbohydrate, and/or carbohydrate-carbohydrate interactions that are essential to maintain diverse cellular processes including pluripotency. On the other hand, it is well known that abnormal glycoprotein expressions can lead to disease pathogenesis^38^,^39^,^40^. In turn, the contributions of these glycoprotein alterations affect both basic research and the clinical use of hiPSCs (Fig. 6). Therefore, it is important to gain knowledge about the glycoprotein alterations and their downstream roles in hiPSCs. In particular, in view of patient-specific hiPSCs, it is essential to understand the functional roles of altered glycoproteins for clinical safety. Here, our integrative omics study not only identified the N-glycoprotein alterations in hiPSCs in comparison to hESCs but also provided evidence of their upstream molecular causes and their downstream functional roles. Our results, together with those of previously published large-scale studies, have potential implications for improving reprogramming and induced pluripotency (Fig. 6). For example, eliminating or significantly reducing the abnormal N-glycoprotein levels and other types of molecular abnormalities in patient-specific hiPSCs may improve the quality of iPS cell-based therapeutics.

Recent reviews^41^,^42^ imply that there are three stages involved in somatic cell reprogramming to induced pluripotency: early, intermediate, and late phases. The early phase is associated with the cellular processes such as the mesenchymal to epithelial transition (MET), resistance to apoptosis, cell proliferation, cell cycle regulation, and metabolic shift. The intermediate and late phases are mainly associated with demethylation of pluripotency gene promotors and transgene independence, respectively. In early phase of hiPSCs, inhibition of MAPK and TGFβ signaling promotes MET, and activation of PI3K-Akt signaling is required for the metabolic shift from oxidative phosphorylation to glycolysis. Our study uncovered that the altered N-glycoprotein expression levels in hiPSCs are involved in a variety of cellular processes and signaling pathways linked with cell adhesion (Fig. 2b,d). Interestingly, the functional network uncovered that the interactions between the altered N-glycoproteins and pluripotency-associated proteins potentially affect the focal adhesion, MAPK signaling, and PI3K-Akt signaling pathways (Fig. 4b and Supplementary Table 4). The focal adhesion pathway plays key roles in cell motility, cell proliferation, and cell survival. Our data suggests that MET, metabolic shift, and cell proliferation are the major targets of N-glycoprotein alterations observed in hiPSCs. These results will be the basis for future studies to determine the detailed mechanism by which altered N-glycoproteins affect the MET process that is coupled with somatic cell reprogramming to induced pluripotency.

Differences in gene expression levels have been identified in hiPSCs compared to hESCs due to incomplete silencing, enhanced silencing, incomplete reactivation, or over-reactivation of the genes during pluripotency reprogramming (i.e., imperfect reprogramming of the genes). These imperfect reprogramming events were described well in a previous study by Ohi and coworkers^14^. This study identified altered gene expression levels of OST complex subunits in hiPSCs compared to hESCs (Fig. 5). These alterations in the OST complex could be one of the potential causes of the N-glycoprotein alterations observed in hiPSCs, as this complex is involved in the protein N-glycosylation process. The other potential causes are cell culture conditions as they can affect metabolic processes (e.g., N-glycan biosynthesis) in both hiPSCs and hESCs. In support of this hypothesis, we observed differences in the gene expression levels of the DPM complex, which is involved in the N-glycan biosynthesis process (Fig. 5). Although cumulative alterations in the OST and DPM complexes cause changes in N-glycoprotein expression levels, we reasoned that increased expression of the catalytic subunit of the OST complex, STT3A, may represent the increased N-glycoprotein expression levels in hiPSCs. It will be interesting to study the activity of STT3A and the changes in N-glycoprotein expression levels during reprogramming process, which is beyond the scope of this study. Conversely, the decreased expression levels of DPM complex subunits observed may lead to decreased N-glycoprotein expression in hiPSCs. Furthermore, nonspecific causes, such as stress during reprogramming, may also result in increased or decreased expression of N-glycoproteins. Therefore, examinations of stress-related genes and pathways could possibly reveal the mechanisms associated with the changes in N-glycoproteins. In an attempt to further elucidate the effectors of the imperfect reprogramming of the OST and DPM complexes, we examined the gene expression levels of the pluripotency-associated core transcription factors (POU5F1, Sox2, and Nanog). Differences were observed in the Sox2 levels between hiPSCs (Gra1 and Gra2) and hESCs (H9 and NTU1), and in the POU5F1 levels between hiPSCs (CBF46 and CBF50) and hESCs (H9 and NTU1) (p < 0.005). We believe that the observed significant alterations in the core transcription factors may affect the expression levels of the OST and DPM complexes. However, it will be worthwhile to elucidate the roles of core transcription factors in regulating the levels of OST and DPM complexes to explore the molecular causes of the alterations in N-glycoprotein expression levels and improve the reprogramming process.

Interestingly, several of the proteins that exhibited alterations in N-glycoprotein expression levels (e.g., EGFR (epidermal growth factor receptor), LGALS3BP (Galectin-3-binding protein) and PLOD3 (Procollagen-lysine, 2-oxoglutarate 5-dioxygenase 3)) in hiPSCs are linked with cancer (Supplementary Fig. 2b and Supplementary Table 2). In support of these results, altered N-glycoproteins were associated with various disease pathways, including cancer (Supplementary Table 4). However, we observed that the N-glycopeptides of several cancer-associated proteins (e.g., EGFR, LGALS3BP, and PLOD3) were down-regulated in hiPSCs compared to hESCs (Supplementary Fig. 2b). Therefore, glycomics analysis in future studies would reveal the details of site-occupancy, such as alterations in glycan structure, which is important to correlate the N-glycan levels with the cancer state. Further analyses using glycosite-directed mutagenesis are critical to determine whether the observed N-glycoprotein alterations were associated with cancer pathogenesis or the initiation of any other disease. In other words, confirmation of the harmlessness/harmfulness of the altered N-glycoproteins observed in hiPSCs is necessary to determine the clinical safety of hiPSC-based therapeutics.

In summary, the current study expands our knowledge of this important class of posttranslational modifications (i.e., N-glycosylated proteins) to improve the cell reprogramming process and induced pluripotency of hiPSCs (Fig. 6). The eventual translation of this knowledge into preclinical studies of hiPSCs will be a remarkable implication of this study.

## Methods

### Cell culture

Human granulosa cells (HGra) were obtained from the *In vitro* Fertilization Center at National Taiwan University (NTU), Taiwan. Human foreskin fibroblasts (HF) were obtained from a 28-year-old male who provided written informed consent in accordance with the protocol approved by the Internal Research Board of NTU Hospital and Academia Sinica, Taiwan. All experimental methods were carried out in accordance with the relevant guidelines and regulations. The Gra1, Gra2, and Gra7 hiPSCs were derived from HGra cells, and the CBF46 and CBF50 hiPSCs were derived from HF cells through a reprogramming process as described in our previous studies^24^,^25^. The H9 cells were obtained from WiCell Research Institute Inc., Madison, WI, USA. NTU1 cells were established as described in our previous study^43^. Somatic cells (HGra and HF) were cultured in RPMI (Invitrogen) supplemented with 10% fetal bovine serum (FBS, Invitrogen), 1% nonessential amino acids (Invitrogen), 2 mM L-glutamine (Invitrogen), and 1% penicillin/streptomycin (Invitrogen). The hESCs (H9 and NTU1) and hiPSCs (Gra1, Gra2, Gra7, CBF46, and CBF50) were grown on MEF feeders (2 × 10^4^ cells/cm^2^) in DMEM/F-12 (Invitrogen) with 20% knock-out Serum Replacement (Invitrogen) and 4 ng/mL bFGF (Sigma).

### Cell lysis, Trypsin digestion, and Sample preparation

Cells were lysed using a modified RIPA buffer and centrifuged (15,000 × g) for 5 min to collect the supernatants. The modified RIPA buffer consists of 1% NP40 (Igepal CA-630), 1 mM EDTA, 0.5 mM DTT, 300 mM NaCl, 10 mM NaF, 1 mM NaVO3, and a protease inhibitor cocktail (Roche, Indianapolis, IN, USA). A previously described standard protocol^21^,^28^ was used for the in-solution trypsin (Promega, Madison, WI) digestion. Briefly, the proteins were denatured using 6 M urea, reduced (10 mM DTT), and alkylated (40 mM IAA). Excess IAA was quenched with 30 mM DTT, and the urea concentration was reduced to 1 M to facilitate optimal trypsin digestion. Then, the proteins were digested with trypsin (protein to trypsin ratio of 50:1) for overnight at 37 °C. The digestion process was stopped by adding formic acid to a final concentration of 0.1%. The tryptic peptide samples were desalted on a C18 column (Waters) to remove the excess urea/salts and then dried in a SpeedVac. The dried samples were subjected to N-glycopeptides enrichment.

### N-glycopeptides enrichment process

Hydrazide chemistry approach was used to enrich the N-glycopeptides as described in a previous study^44^, with modifications. The tryptic peptides of all samples were dissolved in coupling buffer at pH 5.5 (100 mM NaOAc and 150 mM NaCl) and then an oxidizing agent (sodium periodate (15 mM), Sigma-Aldrich, St. Louis, MO) was added to the samples. The reaction mixture was incubated for 30 min in the dark, and the excess oxidant was quenched with sodium sulfite (20 mM) for 10 min. The hydrazide resin (Bio-Rad, Hercules, CA) was added (20 mg/mL) to the peptides solution and subjected to the coupling reaction overnight at 37 °C. Two sequential washings of the hydrazide resin was performed with water, 1.5 M NaCl, MeOH, and 80% ACN. PNGase F obtained from New England Biolabs was used (1 μL/2–6 mg of crude protein) to release the resin bound glycans from N-glycopeptides. The PNGase F reaction was performed by overnight incubation at 37 °C in 100 mM NH4HCO3 solution. Finally, the N-glycopeptides were obtained by collecting the supernatant (A). In addition, the resin was washed with an 80% ACN and supernatant (B) was collected, and then supernatants A and B were mixed. The samples were desalted using a C18 column prior to the LC-MS/MS analysis.

### LC-MS/MS analysis

Each biological sample was analyzed in three technical replicates using LC-MS/MS, as described in our previous studies^21^,^28^. An LTQ-Orbitrap XL ETD mass spectrometer (Thermo Fisher Scientific, San Jose, CA) with a nanoelectrospray ion source (New Objective, Inc.) and an Accela LC system (Thermo Fisher Scientific, San Jose, CA) was used in this study. The electrospray voltage and capillary temperature were set to 1.8 kV and 200 °C, respectively. The 5 μL samples were injected at 10 μL/min flow rate on to a self-packed precolumn (150 μm I.D. × 30 mm, 5 μm, 200 Å). A self-packed reversed phase C18 nanocolumn (75 μm I.D. × 200 mm, 3 μm, 200 Å) was used for online chromatographic separation. We applied 0.1% formic acid in water as mobile phase A and 0.1% formic acid in 80% acetonitrile as mobile phase B. The split flow rate was 300 nL/min. The full-scan mass range was set from m/z 300–2000 with a resolution of 60,000 at m/z 400 to obtain the MS data. The ten most abundant precursor ions were sequentially isolated for MS2 using LTQ.

### MaxQuant analysis of raw data

We processed the MS raw files using MaxQuant tool (version 1.3.0.5) as described previosuly^21^,^26^. The MaxQuant tool consists of an integrated search engine, Andromeda. We performed the MS/MS spectra search against the decoy ipi_HUMAN_3.87 database that consists of 91,464 entries. The parameters such as a precursor mass tolerance of 20 ppm (first search for mass calibration) and of 6 ppm (main search) and a fragment mass tolerance of 0.5 Da (main search) were used in the MaxQuant analysis. We used enzyme trypsin, two missed cleavages, peptide length of seven amino acids, false discovery rate (FDR) of 0.01, variable modifications of oxidation (M) and acetylation (protein N-terminal), and fixed modification of carbamidomethyl (C) as search parameters. In addition, we set the variable modification deamidation (NQ) for the N-glycopeptides identification.

### N-glycoproteomics data analysis

The N-glycoproteomics data obtained from MaxQuant were further processed to perform the multiplexed label-free quantitative analysis. First, the contaminants and reverse identifications were excluded. Furthermore, two filters, score for site localization ≥50 and localization probability ≥0.8 (in ≥2 replicate analyses) were applied. In addition, we only selected the N-glycopeptides with conserved sequence for further analysis. Mean normalization was performed and the N-glycopeptide expression levels with ≤ 20% coefficient of variation (CV) were considered to identify the differentially regulated N-glycoproteins (≥2-fold changes) between the cell lines. The N-glycopeptides with variable (oxidation and acetylation) modifications, which may influence the N-glycopeptide expression levels, were not considered for the identification of differentially regulated events. Here, we utilized proteomics data from our previous study^21^ to calibrate the N-glycoproteomics data. The cell lysates that were used in the current study and in our previous proteomics study were prepared from the same batch of cell pellets. In addition, the proteins were digested using the similar conditions, and the mass spectrometry analysis was performed using the same instrument.

### Microarray data analysis

The microarray data from our previous studies were obtained from the NCBI GEO repository or used directly from our lab. The array data were processed and normalized using GeneSpring 13.1.1 software from Agilent. The probes that exhibited lowest 20% of the range of intensities in all samples were excluded. The probes with highest total intensities were considered in the subsequent analysis to eliminate probe sets redundancy.

### Protein interaction network and enrichment analyses

The functional protein-protein interactions were analyzed using the STRING database (v.10.0a). The STRING database is useful for inputting the proteins of interest and outputting the direct/indirect interactions among the proteins. Here, the STRING analysis was performed by using high confidence (confidence score ≥0.7) and active prediction methods (experiments, databases, and text mining) to generate the protein interaction network. Experiments, databases, and text mining methods indicate that the information is extracted from other protein-protein interaction databases, curated databases, and scientific literature searches, respectively. We then generated 1,000 randomized networks using the Random Network Plugin in Cytoscape^45^. The plugin is available for download on the Random Network Plugin home page. The confidence of the functional network relationships were assessed by comparing its clustering coefficients and mean shortest path values against randomized networks, and the network significance (empirical p-values) values were computed. The Gene Ontology cellular components and biological processes, as well as KEGG pathways of regulated N-glycoproteins were enriched using the DAVID 6.7 database. The KEGG pathways were enriched with the STRING database to identify the canonical pathways in the protein interaction network.

The MS data has been deposited in ProteomeXchange^46^ and the data set identifier is available in additional information.

## Additional Information

**Accession Codes**: The mass spectrometry proteomics data have been deposited to the ProteomeXchange Consortium via the PRIDE partner repository with the dataset identifier PXD004366.

**How to cite this article**: Sudhir, P.-R. *et al*. Integrative omics connects N-glycoproteome-wide alterations with pathways and regulatory events in induced pluripotent stem cells. *Sci. Rep.* **6**, 36109; doi: 10.1038/srep36109 (2016).

**Publisher’s note**: Springer Nature remains neutral with regard to jurisdictional claims in published maps and institutional affiliations.

## Supplementary Material

Supplementary Information

Supplementary Table 1

Supplementary Table 2

Supplementary Table 3

Supplementary Table 4

## References

[b1] Takahashi K. . Induction of pluripotent stem cells from adult human fibroblasts by defined factors. Cell 131, 861–872, doi: 10.1016/j.cell.2007.11.019 (2007). 18035408

[b2] Takahashi K. & Yamanaka S. Induction of pluripotent stem cells from mouse embryonic and adult fibroblast cultures by defined factors. Cell 126, 663–676, doi: 10.1016/j.cell.2006.07.024 (2006). 16904174

[b3] Park I. H. . Reprogramming of human somatic cells to pluripotency with defined factors. Nature 451, 141–146, doi: 10.1038/nature06534 (2008). 18157115

[b4] Jang J. . Disease-specific induced pluripotent stem cells: a platform for human disease modeling and drug discovery. Experimental & molecular medicine 44, 202–213, doi: 10.3858/emm.2012.44.3.015 (2012). 22179105 PMC3317484

[b5] Stadtfeld M. & Hochedlinger K. Induced pluripotency: history, mechanisms, and applications. Genes & development 24, 2239–2263, doi: 10.1101/gad.1963910 (2010). 20952534 PMC2956203

[b6] Ben-David U. & Benvenisty N. The tumorigenicity of human embryonic and induced pluripotent stem cells. Nature reviews. Cancer 11, 268–277, doi: 10.1038/nrc3034 (2011). 21390058

[b7] Mayshar Y. . Identification and classification of chromosomal aberrations in human induced pluripotent stem cells. Cell stem cell 7, 521–531, doi: 10.1016/j.stem.2010.07.017 (2010). 20887957

[b8] Bilic J. & Izpisua Belmonte J. C. Concise review: Induced pluripotent stem cells versus embryonic stem cells: close enough or yet too far apart? Stem cells (Dayton, Ohio) 30, 33–41, doi: 10.1002/stem.700 (2012). 22213481

[b9] Puri M. C. & Nagy A. Concise review: Embryonic stem cells versus induced pluripotent stem cells: the game is on. Stem cells (Dayton, Ohio) 30, 10–14, doi: 10.1002/stem.788 (2012). 22102565

[b10] Narsinh K. H., Plews J. & Wu J. C. Comparison of human induced pluripotent and embryonic stem cells: fraternal or identical twins? Molecular therapy: the journal of the American Society of Gene Therapy 19, 635–638, doi: 10.1038/mt.2011.41 (2011). 21455209 PMC3070108

[b11] Dejosez M. & Zwaka T. P. Pluripotency and nuclear reprogramming. Annual review of biochemistry 81, 737–765, doi: 10.1146/annurev-biochem-052709–104948 (2012). 22443931

[b12] Huang K. . A panel of CpG methylation sites distinguishes human embryonic stem cells and induced pluripotent stem cells. Stem cell reports 2, 36–43, doi: 10.1016/j.stemcr.2013.11.003 (2014). 24511466 PMC3916755

[b13] Hussein S. M. . Copy number variation and selection during reprogramming to pluripotency. Nature 471, 58–62, doi: 10.1038/nature09871 (2011). 21368824

[b14] Ohi Y. . Incomplete DNA methylation underlies a transcriptional memory of somatic cells in human iPS cells. Nature cell biology 13, 541–549, doi: 10.1038/ncb2239 (2011). 21499256 PMC3987913

[b15] Razak S. R. . Profiling of microRNA in human and mouse ES and iPS cells reveals overlapping but distinct microRNA expression patterns. PloS one 8, e73532, doi: 10.1371/journal.pone.0073532 (2013). 24086284 PMC3781120

[b16] Wilson K. D. . MicroRNA profiling of human-induced pluripotent stem cells. Stem cells and development 18, 749–758, doi: 10.1089/scd.2008.0247 (2009). 19284351 PMC3135181

[b17] Chin M. H. . Induced pluripotent stem cells and embryonic stem cells are distinguished by gene expression signatures. Cell stem cell 5, 111–123, doi: 10.1016/j.stem.2009.06.008 (2009). 19570518 PMC3448781

[b18] Ghosh Z. . Persistent donor cell gene expression among human induced pluripotent stem cells contributes to differences with human embryonic stem cells. PloS one 5, e8975, doi: 10.1371/journal.pone.0008975 (2010). 20126639 PMC2813859

[b19] Munoz J. . The quantitative proteomes of human-induced pluripotent stem cells and embryonic stem cells. Molecular systems biology 7, 550, doi: 10.1038/msb.2011.84 (2011). 22108792 PMC3261715

[b20] Phanstiel D. H. . Proteomic and phosphoproteomic comparison of human ES and iPS cells. Nature methods 8, 821–827, doi: 10.1038/nmeth.1699 (2011). 21983960 PMC3432645

[b21] Sudhir P. R. . Quantitative proteomics of protein complexes and their implications for cell reprograming and pluripotency. Journal of proteome research 12, 5878–5890, doi: 10.1021/pr4008877 (2013). 24256468

[b22] Panopoulos A. D. . The metabolome of induced pluripotent stem cells reveals metabolic changes occurring in somatic cell reprogramming. Cell research 22, 168–177, doi: 10.1038/cr.2011.177 (2012). 22064701 PMC3252494

[b23] Boheler K. R. . A human pluripotent stem cell surface N-glycoproteome resource reveals markers, extracellular epitopes, and drug targets. Stem cell reports 3, 185–203, doi: 10.1016/j.stemcr.2014.05.002 (2014). 25068131 PMC4110789

[b24] Huang H. P. . Epithelial cell adhesion molecule (EpCAM) complex proteins promote transcription factor-mediated pluripotency reprogramming. The Journal of biological chemistry 286, 33520–33532, doi: 10.1074/jbc.M111.256164 (2011). 21799003 PMC3190890

[b25] Chuang C. Y. . Granulosa cell-derived induced pluripotent stem cells exhibit pro-trophoblastic differentiation potential. Stem cell research & therapy 6, 14, doi: 10.1186/s13287–015–0005–5 (2015). 25889179 PMC4430911

[b26] Cox J. & Mann M. MaxQuant enables high peptide identification rates, individualized p.p.b.-range mass accuracies and proteome-wide protein quantification. Nature biotechnology 26, 1367–1372, doi: 10.1038/nbt.1511 (2008). 19029910

[b27] Tyanova S. . Visualization of LC-MS/MS proteomics data in MaxQuant. Proteomics 15, 1453–1456, doi: 10.1002/pmic.201400449 (2015). 25644178 PMC5024039

[b28] Sudhir P. R. . Label-free quantitative proteomics and N-glycoproteomics analysis of KRAS-activated human bronchial epithelial cells. Molecular & cellular proteomics: MCP 11, 901–915, doi: 10.1074/mcp.M112.020875 (2012). 22761399 PMC3494152

[b29] Zielinska D. F., Gnad F., Wisniewski J. R. & Mann M. Precision mapping of an *in vivo* N-glycoproteome reveals rigid topological and sequence constraints. Cell 141, 897–907, doi: 10.1016/j.cell.2010.04.012 (2010). 20510933

[b30] Jensen L. J. . STRING 8–a global view on proteins and their functional interactions in 630 organisms. Nucleic acids research 37, D412–D416, doi: 10.1093/nar/gkn760 (2009). 18940858 PMC2686466

[b31] Muller F. J. . Regulatory networks define phenotypic classes of human stem cell lines. Nature 455, 401–405, doi: 10.1038/nature07213 (2008). 18724358 PMC2637443

[b32] Mohorko E., Glockshuber R. & Aebi M. Oligosaccharyltransferase: the central enzyme of N-linked protein glycosylation. Journal of inherited metabolic disease 34, 869–878, doi: 10.1007/s10545-011-9337-1 (2011). 21614585

[b33] Ruepp A. . CORUM: the comprehensive resource of mammalian protein complexes. Nucleic acids research 36, D646–D650, doi: 10.1093/nar/gkm936 (2008). 17965090 PMC2238909

[b34] Ruepp A. . CORUM: the comprehensive resource of mammalian protein complexes–2009. Nucleic acids research 38, D497–D501, doi: 10.1093/nar/gkp914 (2010). 19884131 PMC2808912

[b35] Marchetto M. C. . Transcriptional signature and memory retention of human-induced pluripotent stem cells. PloS one 4, e7076, doi: 10.1371/journal.pone.0007076 (2009). 19763270 PMC2741600

[b36] Liu Y., Cheng D., Li Z., Gao X. & Wang H. The gene expression profiles of induced pluripotent stem cells (iPSCs) generated by a non-integrating method are more similar to embryonic stem cells than those of iPSCs generated by an integrating method. Genetics and molecular biology 35, 693–700, doi: 10.1590/s1415-47572012005000050 (2012). 23055811 PMC3459422

[b37] Wang Y. C., Peterson S. E. & Loring J. F. Protein post-translational modifications and regulation of pluripotency in human stem cells. Cell research 24, 143–160, doi: 10.1038/cr.2013.151 (2014). 24217768 PMC3915910

[b38] Dennis J. W., Granovsky M. & Warren C. E. Glycoprotein glycosylation and cancer progression. Biochimica et biophysica acta 1473, 21–34 (1999). 10580127 10.1016/s0304-4165(99)00167-1

[b39] Pan S., Chen R., Aebersold R. & Brentnall T. A. Mass spectrometry based glycoproteomics–from a proteomics perspective. Molecular & cellular proteomics: MCP 10, R110.003251, doi: 10.1074/mcp.R110.003251 (2011). PMC3013464 20736408

[b40] Pan S. . Quantitative glycoproteomics analysis reveals changes in N-glycosylation level associated with pancreatic ductal adenocarcinoma. Journal of proteome research 13, 1293–1306, doi: 10.1021/pr4010184 (2014). 24471499 PMC3993895

[b41] Apostolou E. & Hochedlinger K. Chromatin dynamics during cellular reprogramming. Nature 502, 462–471, doi: 10.1038/nature12749 (2013). 24153299 PMC4216318

[b42] Hawkins K., Joy S. & McKay T. Cell signalling pathways underlying induced pluripotent stem cell reprogramming. World journal of stem cells 6, 620–628, doi: 10.4252/wjsc.v6.i5.620 (2014). 25426259 PMC4178262

[b43] Chen H. F. . Derivation, characterization and differentiation of human embryonic stem cells: comparing serum-containing versus serum-free media and evidence of germ cell differentiation. Human reproduction (Oxford, England) 22, 567–577, doi: 10.1093/humrep/del412 (2007). 17071820

[b44] Zhang H., Li X. J., Martin D. B. & Aebersold R. Identification and quantification of N-linked glycoproteins using hydrazide chemistry, stable isotope labeling and mass spectrometry. Nature biotechnology 21, 660–666, doi: 10.1038/nbt827 (2003). 12754519

[b45] Shannon P. . Cytoscape: a software environment for integrated models of biomolecular interaction networks. Genome research 13, 2498–2504, doi: 10.1101/gr.1239303 (2003). 14597658 PMC403769

[b46] Vizcaino J. A. . 2016 update of the PRIDE database and its related tools. Nucleic acids research 44, D447–D456, doi: 10.1093/nar/gkv1145 (2016). 26527722 PMC4702828

