# Supplementary material for: Integrative omics connects N-glycoproteome-wide alterations with pathways and regulatory events in induced pluripotent stem cells
Source: Sci Rep. 2016 Nov 3;6:36109. doi: 10.1038/srep36109 (PMC5093713; doi:10.1038/srep36109)
Supplement: Supplementary Information [file srep36109-s1.pdf]

Integrative omics connects N-glycoproteome-wide alterations with pathways and regulatory events in induced pluripotent stem cells

Putty-Reddy Sudhir[1], Madireddy Pavana Kumari[1], Wei-Ting Hsu[2], Chein-Hung Chen[1],
Hung-Chih Kuo[1, 2]*, Chung-Hsuan Chen[1]*
[1]Genomics Research Center and [2] Institute of Cellular and Organismic Biology,
Academia Sinica, Taipei 11529, Taiwan

Supplementary Table Legends

Supplementary Table 1. Master table of N-glycoproteomics analysis of nine cell lines is shown. Five hiPSCs (Gra1, Gra2, Gra7, CBF46, and CBF50) two hESCs (H9 and NTU1), and two SCs (HGra and HF)) were used in this study. The IPI accession numbers and FASTA headers of N-glycoproteins, modified sequence and sequence window of N-glycopeptides, localization score of N-glycosites, normalized abundance that is averaged from three replicate analyzes, and coefficient of variation (CV) of abundance in replicates are shown.

Supplementary Table 2. Lists of altered N-glycoproteins identified in 19 quantitative comparisons are shown. The label-free quantitative analysis was performed between hiPSCs and hESCs as well as between hPSCs and SCs. The IPI accession numbers and FASTA headers of N-glycoproteins, modified sequence and sequence window of N-glycopeptides, N-glycopeptide abundance, coefficient of variation (CV) of abundance, Log 2 ratio are shown.

Supplementary Table 3. Table shows the Gene Ontology (GO) cellular components and biological processes as well as the KEGG pathways linked with hiPSC type-specifically altered N-glycoproteins identified in comparisons of hiPS and hES cells. The GO terms, signaling pathways names, genes involved, p-value, FDR values, Bonferroni values, and Benjamini values are shown.

Supplementary Table 4. KEGG pathways enriched in the protein interaction network are listed. The pathways names, proteins involved, p-value, p-value FDR, p-value Bonferroni are shown.

Supplementary Fig. 1

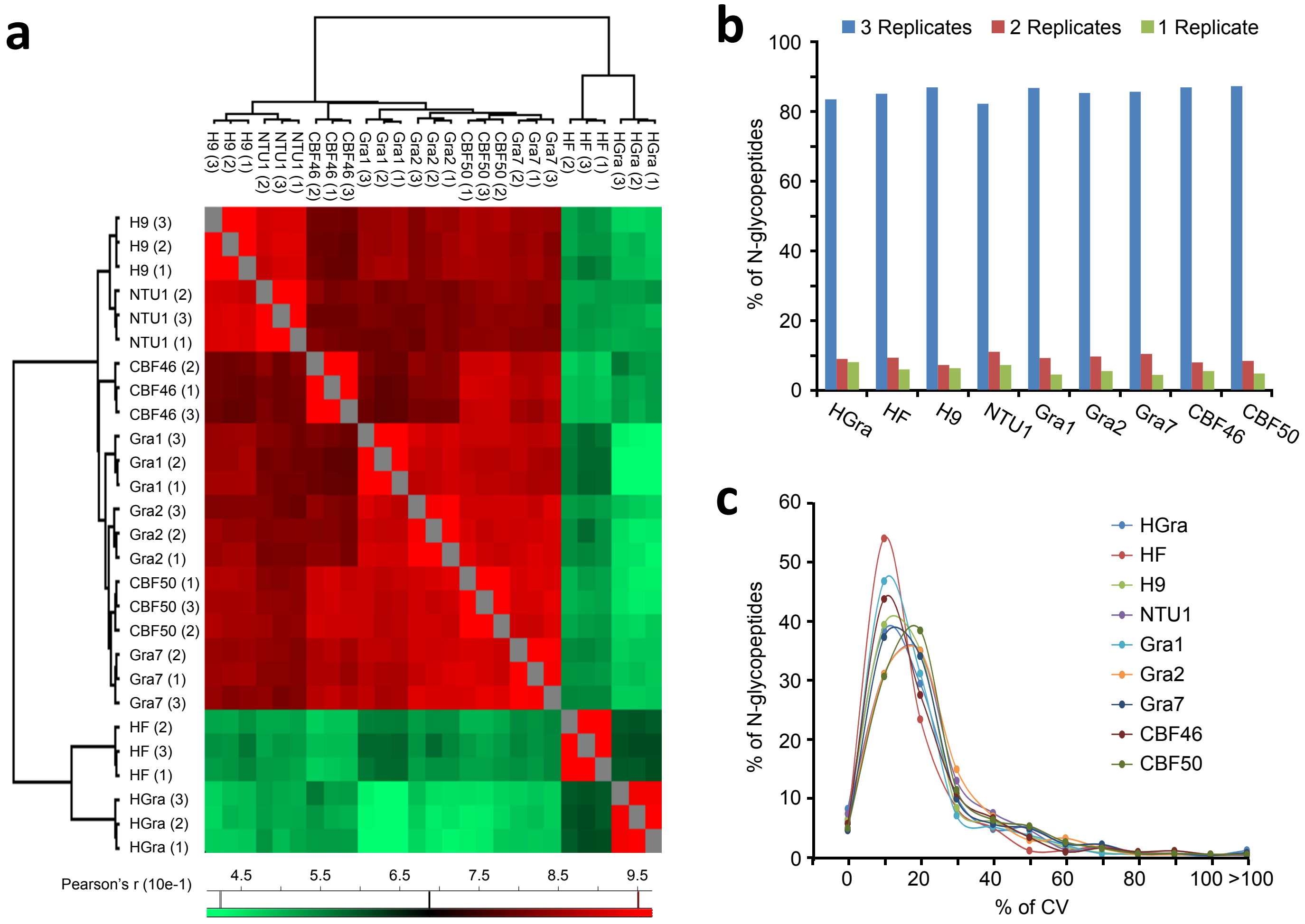

Supplementary Figure 1. Quality assessment of the N-glycoproteomics results. **(a)** Hierarchical clustering analysis of N-glycopeptides expression levels of hiPSCs, hESCs, and SCs. Heat map shows Pearson correlations between 27 replicate analyses from all of the nine cell lines and the color bar represents their Pearson correlation (r) values. **(b)** The percentages of N-glycopeptides identified in three, two, or any one of the replicate analyses are shown for nine cell lines. **(c)** The distribution of N-glycopeptides abundance variation in replicate analyses is shown for nine cell lines by using % of CV (coefficient of variation).

Supplementary Fig. 2

a

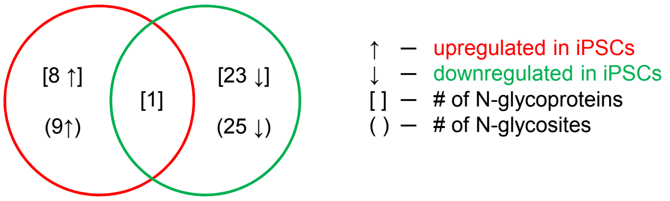

b

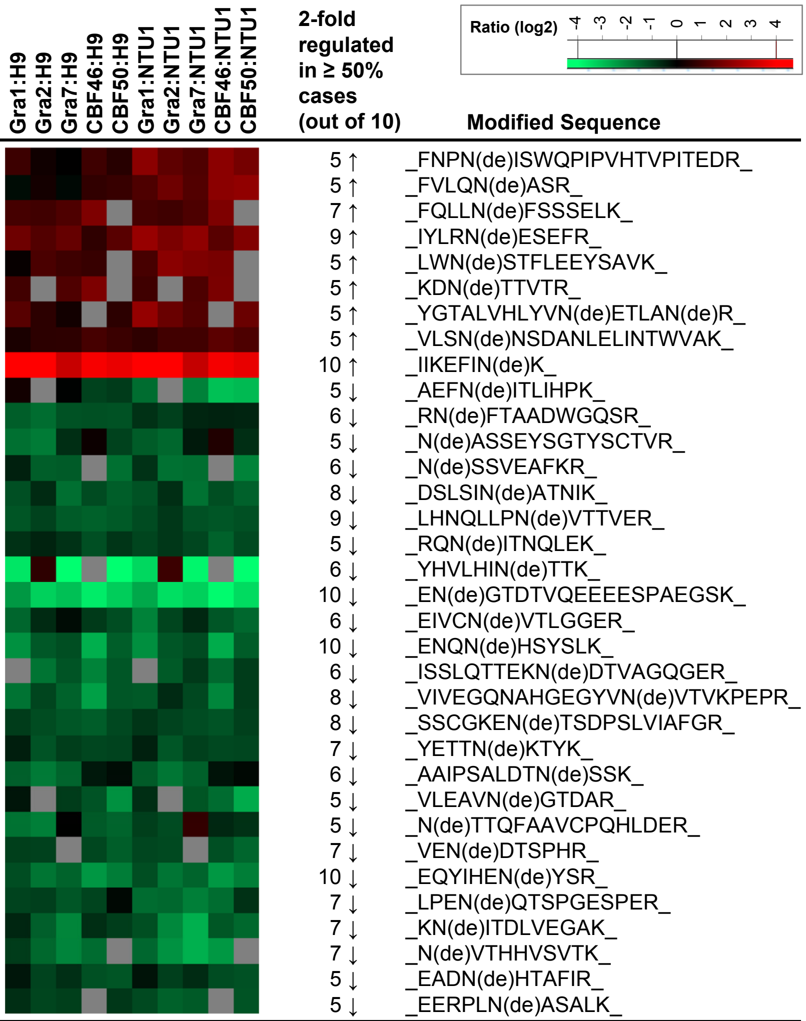

Protein Acc. No.	Gene Symbol	2-fold regulated in ≥ 50% cases (out of 10)	Modified Sequence
IPI00003807	ACP2	5 ↑	_FNPN(de)ISWQPIPVHTVPITEDR_
IPI00015101	C9orf167	5 ↑	_FVLQN(de)ASR_
IPI01012400	CADM1	7 ↑	_FQLLN(de)FSSSELK_
IPI00306604	ITGA5	9 ↑	_IYLRN(de)ESEFR_
IPI00014478	ITGA7	5 ↑	_LWN(de)STFLEEYSAVK_
IPI00884105	LAMP1	5 ↑	_KDN(de)TTVTR_
IPI00872579	PCDH1	5 ↑	_YGTALVHLYVN(de)ETLAN(de)R_
IPI00879931	SERPING1	5 ↑	_VLSN(de)NSDANLELINTWVAK_
IPI00003909	SLC2A3	10 ↑	_IIKEFIN(de)K_
IPI00221224	ANPEP	5 ↓	_AEFN(de)ITLIHPK_
IPI00291136	COL6A1	6 ↓	_RN(de)FTAADWGQSR_
IPI00019146	CXADR	5 ↓	_N(de)ASSEYSGTYSCTVR_
IPI00002478	ECE1	6 ↓	_N(de)SSVEAFKR_
IPI00018274	EGFR	8 ↓	_DSLSIN(de)ATNIK_
IPI00018901	GGT1	9 ↓	_LHNQLLPN(de)VTTVER_
IPI00020470	GLT8D1	5 ↓	_RQN(de)ITNQLEK_
IPI00549891	HS2ST1	6 ↓	_YHVLHIN(de)TTK_
IPI00000877	HYOU1	10 ↓	_EN(de)GTDTVQEEEESPAEGSK_
IPI00031620	ICAM3	6 ↓	_EIVCN(de)VTLGGER_
IPI00027505	ITGAV	10 ↓	_ENQN(de)HSYSLK_
IPI00027505	ITGAV	6 ↓	_ISSLQTTEKN(de)DTVAGQGER_
IPI00472754	KIAA0319L	8 ↓	_VIVEGQNAHGEGYVN(de)VTVKPEPR_
IPI00884105	LAMP1	8 ↓	_SSCGKEN(de)TSDPSLVIAFGR_
IPI00216172	LAMP2	7 ↓	_YETTN(de)KTYK_
IPI00023673	LGALS3BP	6 ↓	_AAIPSALDTN(de)SSK_
IPI00024811	MPZL2	5 ↓	_VLEAVN(de)GTDAR_
IPI00472566	NLGN4X	5 ↓	_N(de)TTQFAAVCPQHLDER_
IPI00414542	PDCD1LG2	7 ↓	_VEN(de)DTSPHR_
IPI00030255	PLOD3	10 ↓	_EQYIHEN(de)YSR_
IPI00006280	SCAP	7 ↓	_LPEN(de)QTSPGESPER_
IPI00293074	SLC44A2	7 ↓	_KN(de)ITDLVEGAK_
IPI00844303	SUN1	7 ↓	_N(de)VTHHVSVTK_
IPI00853149	TMEM131	5 ↓	_EADN(de)HTAFIR_
IPI00023137	TOR1B	5 ↓	_EERPLN(de)ASALK_

c

GO term	# of Genes
integral (or intrinsic) to membrane (GOCC)	MPZL2, SLC44A2, CADM1, GGT1, ACP2, ANPEP, CXADR, KIAA0319L, HS2ST1, GLT8D1, PCDH1, ECE1, SLC2A3, ITGAV, EGFR, ICAM3, SUN1, C9ORF167, PDCD1LG2, SCAP, TMEM131, LAMP1, LAMP2, ITGA5, ITGA7, NLGN4X (26)
cell adhesion or biological adhesion (GOBP)	EGFR, PCDH1, LGALS3BP, MPZL2, CADM1, ITGA5, ITGAV, ICAM3, NLGN4X, ITGA7, COL6A1, CXADR (12)

Supplementary Figure 2. The cell general N-glycoprotein alterations of hiPSCs. **(a)** Venn diagram represents the numbers of hiPS cell general N-glycoprotein alterations (n = 32) and their N-glycosite-specific events (n = 34). These alterations were observed with ≥ 2-fold up- / down-regulation (CV ≤ 20%) in at least 50% cases out of 10 comparisons performed between five hiPSCs and two hESCs. **(b)** The details of accession numbers (IPI ids), symbols, fold changes in 10 comparisons, and site-specific N-glycopeptides are shown for the 32 N-glycoproteins represented in the Venn diagram (Supplementary Fig. 2a). The color bar represents the fold change values. **(c)** Gene Ontology (GO) terms linked with the majority of the 32 N-glycoproteins are shown. The cellular components (GOCC) and biological processes (GOBP) were derived by using DAVID database.
